# Supplementary figures and images for: Using birth-death processes to infer tumor subpopulation structure from live-cell imaging drug screening data
Source: PLoS Comput Biol. 2024 Mar 6;20(3):e1011888. doi: 10.1371/journal.pcbi.1011888 (PMC10947663; doi:10.1371/journal.pcbi.1011888)

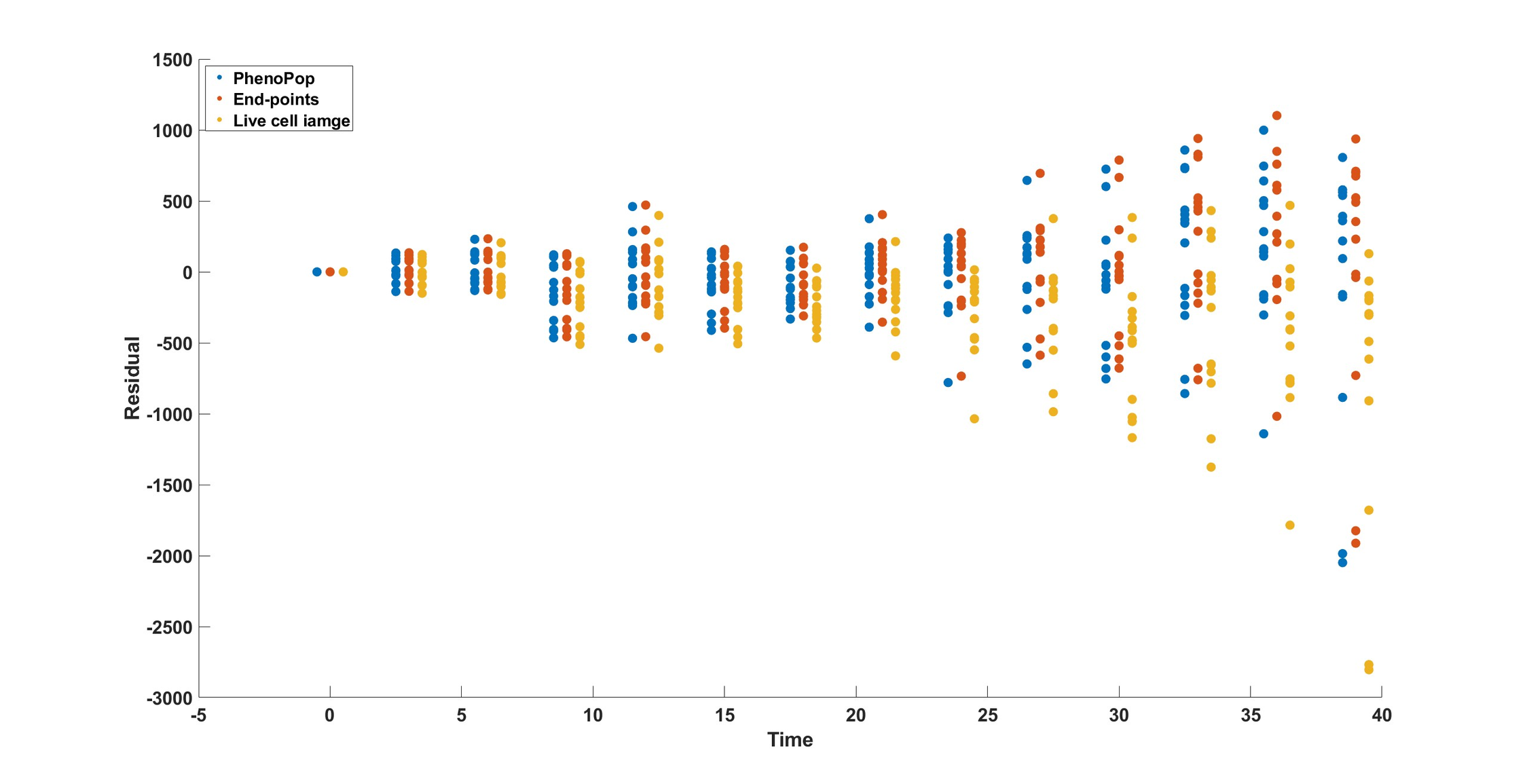

Supplement: S1 Fig — (TIF) [file pcbi.1011888.s001.tif]
