# Supplementary material for: Using birth-death processes to infer tumor subpopulation structure from live-cell imaging drug screening data
Source: PLoS Comput Biol. 2024 Mar 6;20(3):e1011888. doi: 10.1371/journal.pcbi.1011888 (PMC10947663; doi:10.1371/journal.pcbi.1011888)
Supplement: S1 Text — (PDF) [file pcbi.1011888.s002.pdf]

Supporting Information: Using birth-death processes to infer tumor subpopulation structure from live-cell imaging drug screening data

Chenyu Wu[1], Kevin Leder[1*]

1 Department of Industrial and Systems Engineering, University of Minnesota, Minneapolis, MN, United States of America

* lede0024@umn.edu

S1 - Details and extra results of the numerical experiments

We conclude the details setting and some extra results of the numerical experiments.

Generation of simulated data

To simulate data, the parameter set $\theta_{BD}(S)$, as defined in equation (7) of the main text, is selected uniformly at random from a subset of the parameter space given in Table 2 of the main text. Note that one can obtain the generating parameter set $\theta_{PP}(S)$, as defined in equation (2) of the main text, from $\theta_{BD}(S)$ directly by setting $\alpha_i = \beta_i - \nu_i$ for each subpopulation. Based on the parameter set $\theta_{BD}(S)$, we simulated data generated according to the statistical model specified in equation (6) of the main text. Note that data is collected from the simulation continuously during the course of the experiment to replicate the live-cell imaging experiments.

Maximum likelihood estimation (MLE)

The maximum likelihood estimation was conducted by minimizing the negative log-likelihood, subject to constraints that were placed on the range of each parameter. The optimization process to find the minimum point was based on the MATLAB Optimization Toolbox [1] function fmincon with sequential quadratic programming (sqp) solver. Due to the non-convexity of the negative log-likelihood function, we performed the optimization starting from 100 uniformly sampled initial points within a feasible region. The feasible region sets limitations on the parameters based on prior knowledge about them. For simulation studies, the feasible region is given by Table 1, and for the *in vitro* data the feasible region is specified by Table 2. Among all the resulting local optima, the parameter set with the lowest negative log-likelihood is the estimated result.

Bootstrapping

In the simulated experiments, bootstrapping is used to quantify the uncertainty in the MLE estimator. In particular, 20 independent replicates of data measured at 11 concentration values $\mathcal{D}$ and 13 time points $\mathcal{T}$ are generated from the parameters $\theta_{BD}(S)$ at the beginning of the experiment. Then bootstrapping is employed to randomly re-sample 13 replicates from those 20 replicates with replacement 100 times. With 13 randomly sampled replicates it is possible to create an MLE for the parameter set θ_{BD}.

Since there are now 100 MLE's for θ_{BD} it is possible to construct confidence intervals as well by using the empirical quantiles of the estimators.

GR_{50}

Our goal is to estimate the number of subpopulations, initial mixture proportion p_i, and the drug sensitivity of each cellular subpopulation. The GR_{50}, introduced in [2], is a summary metric of drug-sensitivity. It is defined as the concentration at which a drug's effect on cell growth is half the observed effect. Note that at the largest administered concentration level, the drug may not reach its theoretical maximum effect. Therefore, we distinguish GR_{50} from E by noting that GR_{50} represents half the observed maximum effect according to the concentration levels we applied, while E represents half the theoretical maximum effect.

In the context of the model, the GR_{50} can be defined as below. Denote the maximum dosage applied as d_m, and define the half-maximum effect for subpopulation i as $r_i = (\nu_i(d_m) + \nu_i(0))/2$. The explicit formula for the GR_{50} is then for subpopulation i:

$$GR_{50} = E_i \left(\frac{1 - e^{\nu_i - r_i}}{e^{\nu_i - r_i} - b_i} \right)^{1/m_i}$$

When $S = 2$, we will denote the higher GR_{50} as either the resistant GR_{50} or GR_r, and the lower GR_{50} as either the sensitive GR_{50} or GR_s. In this setting, parameters for the sensitive subpopulation and the resistant subpopulation, respectively, are denoted by subscripts s and r, e.g. E_s and E_r.

Initial conditions.

The initial number of cells is set as $n = 1000$, and the initial size of each subpopulation is set by rounding np_i to the nearest integer for subpopulation i. The following drug concentration levels are used

$$\mathcal{D} = [0, 0.0313, 0.0625, 0.1250, 0.2500, 0.3750, 0.5, 1.25, 2.5, 3.75, 5]$$

and we collect the cell count data at the time points:

$$\mathcal{T} = [0, 3, 6, 9, 12, 15, 18, 21, 24, 27, 30, 33, 36].$$

For these specific concentration levels and time points, we have chosen the threshold values of $T_L = 21$ and $D_L = 1$ in the PhenoPop model.

Optimization feasible region

When performing numerical optimization the parameters are restricted to a physically realistic region. Unless otherwise noted, the optimization was performed using 100 uniformly sampled initial points from Table 1.

	p_s	p_r	$\beta_{s,r}$	$\nu_{s,r}$	$b_{s,r}$	$E_{s,r}$	$m_{s,r}$	σ_L, σ_H	c
Range	$[0, 0.5]$	$1 - p_s$	$[0, 1]$	$[\beta - 0.1, \beta]$	$[0.27, 1]$	$[0, 10]$	$[0.01, 10]$	$[0, 2500]$	$[0, 10]$

S1 Table 1. Feasible interval for each parameter.

Estimation on *in vitro* experimental data

When solving the maximum likelihood optimization problems for the *in vitro* data, the optimization feasible region was chosen to be the same as the feasible region used in paper [3] for the *Ba/F3* data, i.e., We solved each optimization problem 500 times

	p	β	ν	b	E	m	σ_L, σ_H	c
Range	$[0,1]$	$[0,1]$	$[\beta - 0.06, \beta]$	$[0.878, 1]$	$[0, 50]$	$[0.001, 20]$	$[0, 2500]$	$[0, 100]$.

S1 Table 2. Optimization Feasible region

starting from randomly chosen initial points.

Imatinib sensitive and resistant *Ba/F3* data estimation residual

To visualize how three methods mentioned in this manuscript fit the imatinib sensitive and resistant *Ba/F3* data, we plotted the residual from the estimated mean behavior for all three methods in Fig. We can clearly see the estimation residual increase as time increases, and thus the need for a model with variance that changes with time.

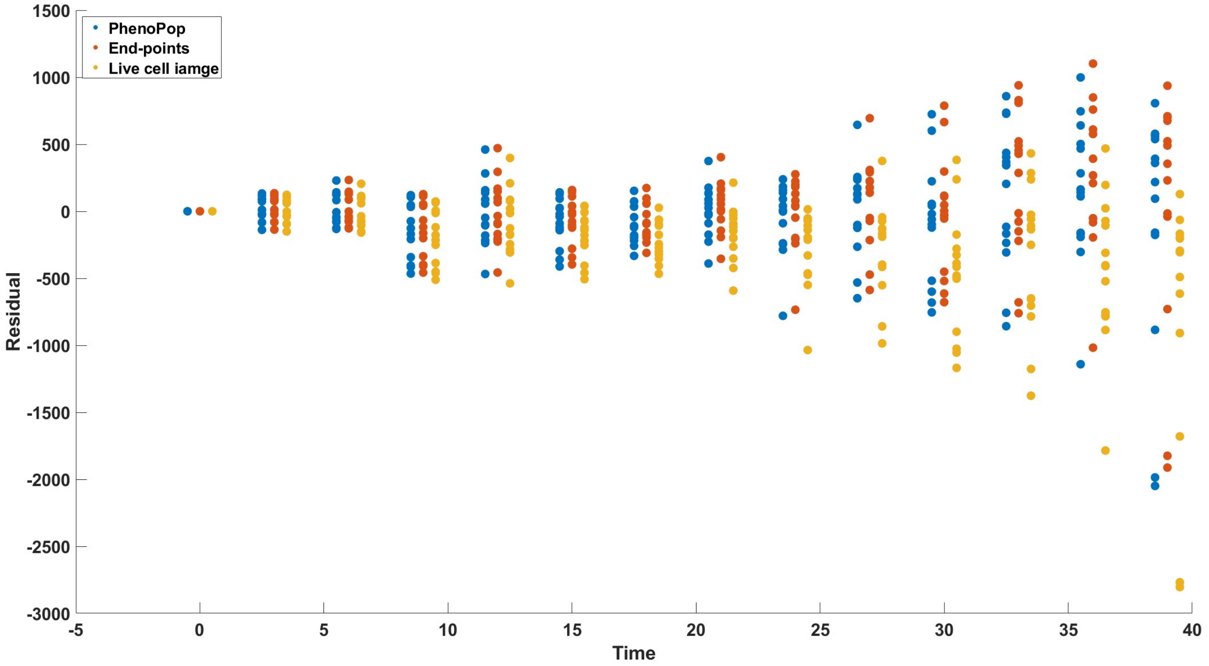

S1 Fig. Scatter plots between time and residual from the mean behavior of all three methods. The range of residual increase with respect to time corresponds to our observation about the increasing variance over time.

Data and code availability

All data and code used for running experiments, model fitting, and plotting are available on a GitHub repository at `https://github.com/chenyuwu233/PhenoPop_stochastic`. The required Matlab version is Matlab R2022a or newer.

References

1. Optimization toolbox version: 23.2 (R2022a), Natick, MA: The MathWorks.
2. Hafner M, Niepel M, Chung M, and Sorger PK. Growth rate inhibition metrics correct for confounders in measuring sensitivity to cancer drugs. *Nature Methods*, 13(6):521–527, October 2015 2016.
3. Köhn-Luque A, Myklebust EM, Tadele DS, Giliberto M, Schmiester L, Noory J, et al. Phenotypic deconvolution in heterogeneous cancer cell populations using drug-screening data. *Cell Reports Methods*, page 100417, 2023.
