# Supplementary material for: Using birth-death processes to infer tumor subpopulation structure from live-cell imaging drug screening data
Source: PLoS Comput Biol. 2024 Mar 6;20(3):e1011888. doi: 10.1371/journal.pcbi.1011888 (PMC10947663; doi:10.1371/journal.pcbi.1011888)
Supplement: S2 Text — (PDF) [file pcbi.1011888.s003.pdf]

Supporting Information: Using birth-death processes to infer tumor subpopulation structure from live-cell imaging drug screening data

Chenyu Wu[1], Kevin Leder[1*]

1 Department of Industrial and Systems Engineering, University of Minnesota, Minneapolis, MN, United States of America

* lede0024@umn.edu

S2 - Proof and extension of proposition 2

Given a set of time points $\mathcal{T} = \{t_1, \cdots, t_k\}$, we first show for any $t_j, 1 \leq j \leq k$ that:

$$W_n(t_j) \Rightarrow Y(t_j) = \sum_{\ell=1}^{j} \sum_{g=1}^{S} \sqrt{p_g} e^{\lambda_g (t_j - t_\ell)} e^{\lambda_g t_{\ell-1}/2} V_g(t_\ell - t_{\ell-1}) \text{ as } n \to \infty,$$

where $V_g(t)$ is a random variable that has normal distribution $N(0, \sigma_g^2(t))$ and the $\sigma_g^2(t)$ here is the variance of subpopulation g linear birth-death process defined in equation (5) of the main text. Since we assume that each cell grows independently, we define

$$X_g(t) = \sum_{m=1}^{X_g(0)} B_g^{(m)}(t), g \in \{1, \cdots, S\},$$

where $B_g^{(m)}(t)$ is the number of descendants generated from the m-th type g cell. This means that $\{B_g^{(m)}; t \geq 0, m \in \{1, \ldots X_g(0)\}$ is an independent sequence of identically distributed linear birth-death processes. Note that due to the Markov property of the linear birth-death process, we can write a more general equation for $0 \leq t_1 \leq t_2$

$$X_g(t_2) = \sum_{m=1}^{X_g(t_1)} B_g^{(m)}(t_2 - t_1).$$

Following an argument from Either and Kurtz [1], we have the decomposition

$$\begin{aligned}
W_n(t_j) &= \frac{1}{\sqrt{n}} \sum_{g=1}^{S} \sum_{\ell=1}^{j} e^{\lambda_g (t_j - t_\ell)} X_g(t_\ell) - e^{\lambda_g (t_j - t_{\ell-1})} X_g(t_{\ell-1}) \\
&= \sum_{\ell=1}^{j} \sum_{g=1}^{S} \frac{1}{\sqrt{n}} e^{\lambda_g (t_j - t_\ell)} \left[X_g(t_\ell) - e^{\lambda_g (t_\ell - t_{\ell-1})} X_g(t_{\ell-1}) \right] \\
&= \sum_{\ell=1}^{j} \sum_{g=1}^{S} \left\{ e^{\lambda_g (t_j - t_\ell)} \left(\frac{p_g n}{n} \right)^{1/2} \left(\frac{X_g(t_{\ell-1})}{p_g n} \right)^{1/2} X_g(t_{\ell-1})^{-1/2} \right. \\
&\quad \left. \sum_{m=1}^{X_g(t_{\ell-1})} \left[B_g^{(m)}(t_\ell - t_{\ell-1}) - e^{\lambda_g (t_\ell - t_{\ell-1})} \right] \right\}
\end{aligned}$$

By assuming the initial proportion p_g for sub-type g is independent of n and using the Law of large numbers, as $n \to \infty$, we have

$$\left(\frac{X_g(t_{\ell-1})}{p_g n}\right) \to \mathbb{E}[B_g^{(m)}(t_{\ell-1})] = e^{\lambda_g t_{\ell-1}} \quad a.s.$$

By assuming that the maximum number of time point N_T and the length of time interval $t_i - t_j$ for any $i \geq j$ are both bounded and not depend on n, the Law of large numbers also assures that for any $\ell \in \{1, \cdots, N_T\}$ the $X_g(t_{\ell-1})$ will diverge to infinity when $n \to \infty$. Therefore, we may apply the Central Limit Theorem to the following term:

$$X_g(t_{\ell-1})^{-1/2} \sum_{m=1}^{X_g(t_{\ell-1})} \left[B_g^{(m)}(t_\ell - t_{\ell-1}) - e^{\lambda_g(t_\ell - t_{\ell-1})}\right] \Rightarrow V_g(t_\ell - t_{\ell-1}) \sim N(0, \sigma_g^2(t_\ell - t_{\ell-1})).$$

Thus, we conclude that

$$W_n(t_j) \Rightarrow \sum_{\ell=1}^{j} \sum_{g=1}^{S} \sqrt{p_g} e^{\lambda_g(t_j - t_\ell)} e^{\lambda_g t_{\ell-1}/2} V_g(t_\ell - t_{\ell-1}) \text{ as } n \to \infty.$$

Next we show that the random vector $\mathbf{W}$ converges to the random vector $\mathbf{Y}$, which has the multivariate normal distribution. We can obtain the distribution for $\mathbf{Y}$ from the independence between $V_i(t_\ell - t_{\ell-1})$ and $V_j(t_m - t_{m-1})$ for all $i, j \in \{1, \cdots, N_g\}, \ell, m \in \{1, \cdots, k\}$:

$$\mathbf{Y} = [Y(t_1), \cdots, Y(t_k)] \sim N(0, \Sigma),$$

where

$$\Sigma_{i,j} = \sum_{\ell=1}^{\min(i,j)} \sum_{g=1}^{S} p_g e^{\lambda_g(t_i - t_\ell)} e^{\lambda_g(t_j - t_\ell)} e^{\lambda_g t_{\ell-1}} \sigma_k^2(t_\ell - t_{\ell-1}).$$

Then we use the Cramer Wold device, given a constant vector $a \in \mathbb{R}^k < \infty$, we have

$$\begin{aligned} \langle a, \mathbf{W} \rangle &= \sum_{j=1}^{k} a_j \sum_{\ell=1}^{j} \sum_{g=1}^{S} \left\{ \sqrt{p_g} e^{\lambda_g(t_j - t_\ell)} \left(\frac{X_g(t_{\ell-1})}{p_g n}\right)^{1/2} X_g(t_{\ell-1})^{-1/2} \right. \\ &\quad \left. \sum_{m=1}^{X_g(t_{\ell-1})} \left[B_g^{(m)}(t_\ell - t_{\ell-1}) - e^{\lambda_g(t_\ell - t_{\ell-1})}\right] \right\} \\ &\Rightarrow \sum_{j=1}^{k} a_j \sum_{\ell=1}^{j} \sum_{g=1}^{S} \sqrt{p_g} e^{\lambda_g(t_j - t_\ell)} e^{\lambda_g t_{\ell-1}/2} V_g(t_\ell - t_{\ell-1}) \\ &= \sum_{j=1}^{k} a_j Y(t_j) = \langle a, \mathbf{Y} \rangle \end{aligned}$$

Thus, we show that $\mathbf{W} \Rightarrow \mathbf{Y}$.

Extension of proposition 2 while initial proportions can go to 0 with $n \to \infty$

In proving Proposition 2, we made the assumption that the initial proportions p_i for $i = 1, \cdots, S$ are not dependent on the initial cell count n. In this sub-section, we aim to

relax this assumption and demonstrate a similar result. Note we will assume that all other inputs are still independent of n, i.e., S and $\mathcal{T}$.

In particular, we will allow p_i to depend on n, and allow for $\limsup_n p_i n < \infty$. We denote two sets of subpopulations F and I, where

$$\begin{aligned} F &= \{i \in \{1, \cdots, S\}; \limsup_{n\to\infty} p_i n < \infty\} \\ I &= \{i \in \{1, \cdots, S\}; \lim_{n\to\infty} p_i n = \infty\} \end{aligned}$$

Due to $\sum_{i=1}^{S} p_i = 1$, and S being fixed with n, we know that the set I must not be an empty set. Then following a similar pattern as the proof of the Proposition 2, we derive:

$$\begin{aligned} W_n(t_j) &= \sum_{\ell=1}^{j}\sum_{g=1}^{S}\Bigg\{ e^{\lambda_g(t_j-t_\ell)}\left(\frac{p_g n}{n}\right)^{1/2}\left(\frac{X_g(t_{\ell-1})}{p_g n}\right)^{1/2} X_g(t_{\ell-1})^{-1/2} \\ &\qquad \sum_{m=1}^{X_g(t_{\ell-1})}\left[B_g^{(m)}(t_\ell - t_{\ell-1}) - e^{\lambda_g(t_\ell - t_{\ell-1})}\right]\Bigg\} \\ &= \sum_{\ell=1}^{j}\sum_{g\in I}\Bigg\{ e^{\lambda_g(t_j-t_\ell)}\left(\frac{p_g n}{n}\right)^{1/2}\left(\frac{X_g(t_{\ell-1})}{p_g n}\right)^{1/2} X_g(t_{\ell-1})^{-1/2} \\ &\qquad \sum_{m=1}^{X_g(t_{\ell-1})}\left[B_g^{(m)}(t_\ell - t_{\ell-1}) - e^{\lambda_g(t_\ell - t_{\ell-1})}\right]\Bigg\} \\ &\quad + \sum_{\ell=1}^{j}\sum_{g\in F}\Bigg\{ e^{\lambda_g(t_j-t_\ell)}\left(\frac{p_g n}{n}\right)^{1/2}\left(\frac{X_g(t_{\ell-1})}{p_g n}\right)^{1/2} X_g(t_{\ell-1})^{-1/2} \\ &\qquad \sum_{m=1}^{X_g(t_{\ell-1})}\left[B_g^{(m)}(t_\ell - t_{\ell-1}) - e^{\lambda_g(t_\ell - t_{\ell-1})}\right]\Bigg\} \end{aligned}$$

Next, we may consider these two double sums separately. For $g \in I$, because the $p_g n$ diverges to infinity as $n \to \infty$, the first double sum will converge to the same limit we established in Proposition 2. For $g \in F$, $p_g n$ will stay bounded. Therefore, in the second double sum, we will have $\frac{p_g n}{n}$ converge to 0 as $n \to \infty$, which makes the second double sum vanish. In conclusion, we have

$$W_n(t_j) \Rightarrow \sum_{\ell=1}^{j}\sum_{g\in I}\sqrt{p_g}\, e^{\lambda_g(t_j-t_\ell)} e^{\lambda_g t_{\ell-1}/2} V_g(t_\ell - t_{\ell-1}) \text{ as } n \to \infty.$$

Note that this convergence result will lead to the same realization in practice, i.e., we will define the covariance by summing over all subtypes. This is because in practice we only have $n < \infty$ and we cannot actually assume subpopulations have zero contribution to the covariance.

References

1. Ethier SN and Kurtz TG. *Markov processes: characterization and convergence.* John Wiley & Sons, 2009.
