# Supplementary material for: Using birth-death processes to infer tumor subpopulation structure from live-cell imaging drug screening data
Source: PLoS Comput Biol. 2024 Mar 6;20(3):e1011888. doi: 10.1371/journal.pcbi.1011888 (PMC10947663; doi:10.1371/journal.pcbi.1011888)
Supplement: S3 Text — (PDF) [file pcbi.1011888.s004.pdf]

Supporting Information: Using birth-death processes to infer tumor subpopulation structure from live-cell imaging drug screening data

Chenyu Wu[1], Kevin Leder[1*]

1 Department of Industrial and Systems Engineering, University of Minnesota, Minneapolis, MN, United States of America

* lede0024@umn.edu

S3 - Exact path likelihood computation

Here we show how to calculate the exact likelihood of a sample path observation of the total cell count of multiple heterogeneous birth-death processes. While we do not use this approach for likelihood evaluation in the current manuscript we report it here to show that it is not feasible. We say an algorithm has computational complexity $\Theta(g(x))$ if there exists positive constants c and C such that computational complexity is greater than $cg(x)$ for all x and less than $Cg(x)$ for all x.

We first consider the following joint probability of a homogeneous linear birth-death process:

$$\begin{aligned} & \mathbb{P}(X(t_1) = x_1, \cdots, X(t_k) = x_k | X(t_0) = n, \theta_{BD}(2)) \\ = & \prod_{k=1}^{N_t} \mathbb{P}(X(t_k) = x_k | X(t_{k-1}) = x_{k-1}, \theta_{BD}(2)). \end{aligned}$$

For ease of notation define the transition probability

$$p_{i,j}(t_k - t_{k-1}) = \mathbb{P}(X(t_k) = j | X(t_{k-1}) = i, \theta_{BD}(2)).$$

It is important to note that evaluating $p_{i,j}(t_k - t_{k-1})$ is the most computation-demanding task when evaluating the joint probability. As a result, we mainly consider the number of evaluations of this transition probability. The analytical form for this transition probability was derived in [1]:

$$p_{i,j}(t) = \sum_{k=0}^{\min(i,j)} \binom{i}{k} \binom{i+j-k-1}{i-1} a(t)^{i-k} b(t)^{j-k} (1 - a(t) - b(t))^k, \tag{1}$$

where

$$a(t) = \frac{\nu(e^{(\beta-\nu)t} - 1)}{\beta e^{(\beta-\nu)t} - \nu}, b(t) = \frac{\beta(e^{(\beta-\nu)t} - 1)}{\beta e^{(\beta-\nu)t} - \nu}.$$

Note that numerical evaluation (1) will be computationally expensive due to the presence of multiple factorial terms. We use a Gosper refined version of the Stirling formula [2] to approximate these factorials

$$n! \approx \sqrt{\left(2n + \frac{1}{3}\right)\pi n^n e^{-n}}.$$

We find that this approximation leads to good performance in our examples. It is thus straightforward to evaluate the path likelihood for the case of a homogeneous linear birth-death process.

If we instead have observations of a sum of birth-death processes, the evaluation of the path likelihood is much more difficult. In particular, the sum of the birth-death processes is no longer a Markov process and we must therefore sum over possible values of our unobserved subpopulations. Specifically, if we have two subpopulations we can formulate the equation (11) of the main text, as

$$\begin{aligned}
&P(X^{(r)}(t) = x_t, t \in \mathcal{T} | \theta_{BD}(2)) \\
&= \sum_{i_1=0}^{x_1} \cdots \sum_{i_{N_T}=0}^{x_{N_T}} \\
&\quad P\left(X_1(t_1) = i_1, X_2(t_1) = x_1 - i_1, \ldots, X_1(t_{N_T}) = i_{N_T}, X_2(t_{N_T}) = x_{N_T} - i_{N_T} | \theta_{BD}(2)\right) \\
&= \sum_{i_1=0}^{x_1} \cdots \sum_{i_{N_T}=0}^{x_{N_T}} \left\{ P\left(X_1(t_1) = i_1, X_1(t_2) = i_2, \cdots, X_1(t_{N_T}) = i_{N_T} | \theta_{BD}(2)\right) \right. \\
&\quad \left. P\left(X_2(t_1) = x_1 - i_1, X_2(t_2) = x_2 - i_2, \ldots, X_2(t_{N_T}) = x_{N_T} - i_{N_T} | \theta_{BD}(2)\right) \right\}.
\end{aligned} \tag{2}$$

Note that the last equality is due to the assumption that subpopulations grow independently, and we can decompose the joint probability of mixture cell count into a summation of multiple joint probabilities of the homogeneous linear birth-death process. It is not hard to see that if we naively evaluate the above sum, the number of computations of the homogeneous joint probability is around $\Theta(x_t^{N_T})$. Since many examples have $N_T \approx 10$, and $x_t \approx 10^3$ this is clearly an infeasible approach.

In order to avoid the exponential dependence on the number of time points, one option is to use techniques from Hidden Markov Models (HMM). The main assumption of HMM is the Markov property of the hidden process, and that the hidden process relates to the observable process according to a specified distribution B. Recall that the time series of observed total cell count is given by $\{X(t_i); i \in \mathcal{T}\}$ and denote the time series of the subpopulations as $\{(X_1(t_i), \ldots, X_S(t_i); i \in \mathcal{T}\}$. Then $\{X(t_i); i \in \mathcal{T}\}$ in the HMM is the observable process, and $\{(X_1(t_i), \ldots, X_S(t_i)); i \in \mathcal{T}\}$ is the hidden Markov process due to the Markov property of the linear birth-death process. Notice that the relationship between the hidden process $\{(X_1(t_i), \ldots, X_S(t_i)); i \in \mathcal{T}\}$ and the observable process $\{X(t_i); i \in \mathcal{T}\}$ can be defined as

$$\mathbb{P}(X(t) = x | (X_1(t), \ldots, X_S(t)) = (x_1, \ldots, x_S)) = \begin{cases} 1 & \text{if } x_1 + \ldots + x_S = x \\ 0 & \text{o.w.} \end{cases}$$

We can translate the live-cell imaging experiment into an HMM and significantly improve the computational complexity of evaluating the exact likelihood function, i.e. equation (8) of the main text. In particular, we can use popular HMM techniques, such as the forward-backward procedure to reduce the total number of transition probability, i.e., equation (18) of the main text, computation to $\Theta(H^2 N_T)$ for one replicate at one dosage level, where H is the number of hidden states. In particular, we need to calculate the H by H transition matrix for every time point, and if we assume the

length of time intervals are identical, we can reduce the upper bound to $\Theta(H^2)$. The number of hidden states depends on both the number of cells observed at each time point x_t and the number of subpopulations S; note that due to the exponential growth, x_t may change drastically as t increases. As we will now show, this is unfortunately not a sufficient reduction in computational complexity. In particular, assume we observe x total cells. In that case, the number of hidden states is given by $\binom{x-1}{S-1}$, and assuming that $x \gg S$, we have that $\binom{x-1}{S-1} = \Theta\left(x^{S-1}\right)$ as $x \to \infty$. With only two subpopulations this results in computational complexity of $\Theta(x^2)$, and in many experiments, we might have maximum total cell number x approximately 10^4 thus requiring approximately 10^8 operations for one likelihood evaluation. If $S = 3$ we would end up with the far worse computational complexity of $\Theta(x^4)$.

In conclusion, using a naive approach to compute the joint probability of mixture cell count would require $\Theta\left(x_t^{N_T}\right)$ many computations of transition probability, which is clearly infeasible when many cell counts are in the thousands with over 10 observed time points. We also discuss an HMM based approach to evaluating the likelihood that results in a significant reduction in the computational burden for evaluating this likelihood. In particular, with this approach we can reduce the number of computations of the transition probability to $\Theta\left(x^2\right)$ with x approximately 10^4. Note that this will be the complexity for evaluating the likelihood of one single replicate at a single dose, so taking into account that we can have more than 10 different doses, with more than 10 replicates at each dose we see that unfortunately, this HMM approach is computationally infeasible. In addition, this HMM approach will have significantly worse computational complexity after we include observation noise terms and/or more than 2 subpopulations.

References

1. Bailey NTJ. *The elements of stochastic processes with applications to the natural sciences*, volume 25. John Wiley & Sons, 1991.

2. Gosper Jr RW. Decision procedure for indefinite hypergeometric summation. *Proceedings of the National Academy of Sciences*, 75(1):40–42, 1978.
